# A KAP model-based retrospective study on the association between fertility stress and marital adjustment in patients undergoing assisted reproductive technology

**DOI:** 10.1097/MD.0000000000044926

**Published:** 2026-01-09

**Authors:** Limin He, Qian Yang, Lian Liu, Lihua Zhou

**Affiliations:** a Department of VIP Clinic Nursing, West China Second University Hospital, Sichuan University/West China School of Nursing, Sichuan University, Chengdu, Sichuan Province , China; b Key Laboratory of Birth Defects and Related Diseases of Women and Children (Sichuan University), Ministry of Education, Chengdu, Sichuan Province, China; c Department of Blood Purification Center, Anyue County People’s Hospital, Anyue , Sichuan Province, China.

**Keywords:** assisted reproductive technology, fertility stress, KAP model, marital adjustment, regression analysis

## Abstract

Patients undergoing assisted reproductive technology (ART) often experience considerable fertility-related stress, which may adversely affect their marital adjustment. The Knowledge–Attitude–Practice (KAP) model provides a theoretical framework for understanding patient cognition and behavior. This study aimed to investigate the relationship between fertility-related stress and marital adjustment, as well as its influencing factors. A retrospective analysis was conducted on 200 ART patients, divided into a high-stress group (n = 100) and a low-stress group (n = 100) based on the median fertility stress score. KAP model scores and marital adjustment levels were compared between groups. Pearson correlation analysis, multivariate linear regression, and subgroup analysis by educational level were employed. The high-stress group had significantly lower scores across all KAP dimensions – knowledge (68.5 ± 9.2 vs 75.3 ± 8.6), attitude (72.1 ± 10.4 vs 78.9 ± 9.1), and practice (65.7 ± 11.0 vs 71.8 ± 10.2) – as well as lower marital adjustment scores (82.3 ± 12.5 vs 91.7 ± 11.3; all *P* < .001). Fertility stress showed a significant inverse association with marital adjustment (*r* = –0.48; β=–0.37; *P* < .001), while higher knowledge and practice scores were independent positive predictors (both *P* < .01). Subgroup analysis indicated a stronger negative correlation among patients with higher education (*r* = –0.52 vs –0.39). These findings suggest not only statistical but also practical significance, as stress reduction and enhancement of knowledge and behavioral practice may directly contribute to better marital adaptation in clinical settings. Higher fertility-related stress is associated with poorer marital adjustment in ART patients. Marital adaptation is closely linked to KAP model components. Greater attention should be given to highly educated individuals, with targeted interventions to improve knowledge and practice capacities to alleviate stress and enhance marital adjustment.

## 1. Introduction

With the accelerating pace of modern life and the increasing delay in childbearing age, the incidence of infertility has been rising steadily in recent years. According to statistics from the World Health Organization, infertility has become a major global public health issue affecting reproductive health, with a prevalence rate of approximately 10% to 15%.^[1,2]^ Assisted reproductive technology (ART) has emerged as a crucial clinical intervention for infertility. In China, the application of ART has rapidly expanded in recent years, enabling a growing number of infertile couples to conceive through in vitro fertilization, intrauterine insemination (IUI), and other methods.^[3]^ However, ART is often associated with long treatment cycles, limited success rates, high financial burdens, and substantial social and emotional pressure. These challenges collectively impose a significant psychological burden on patients, making fertility-related stress a common and critical concern among those undergoing ART.^[4]^ Numerous studies have shown that fertility stress not only affects patients’ emotional well-being and adherence to treatment but also undermines marital relationships, potentially disrupting the entire family system.^[5–8]^ As such, marital adjustment – an important psychosocial indicator of relationship quality – has attracted increasing scholarly attention in the context of ART.

Marital adjustment refers to an individual’s overall adaptability to emotional, communicative, and role-based aspects of marital life. It is closely associated with the quality and stability of marital and family functioning.^[9]^ Previous studies have noted that fertility stress during ART often correlates with elevated anxiety, depression, guilt, and insecurity, which in turn disrupt emotional intimacy and communication between partners. These dynamics can lead to reduced marital satisfaction, frequent conflict, and, in severe cases, marital breakdown.^[10–12]^ For example, research has demonstrated that women undergoing ART report significantly lower marital satisfaction compared to those who conceive naturally, with perceived stress strongly linked to strained marital interactions.^[13]^ Domestic studies have also highlighted that the psychological experiences of individuals undergoing ART not only influence treatment outcomes but also serve as important mediators in marital dynamics.^[14–16]^ Nevertheless, existing research often treats fertility stress and marital adjustment as independent variables, lacking systematic exploration of their interrelationships, particularly under the guidance of a coherent theoretical framework.

The Knowledge–Attitude–Practice (KAP) model, a widely used framework in health education and behavioral science, posits that individuals’ health behaviors are shaped by their level of knowledge, attitudinal orientation, and behavioral competence.^[17,18]^ In recent years, the KAP model has been applied to chronic disease management, psychological interventions, and perinatal health behavior research. Among infertile patients, the KAP model is a useful tool to assess their understanding of ART, emotional responses during treatment, and coping behaviors in daily life.^[19]^ Prior studies have indicated that improving KAP levels in ART patients can alleviate psychological distress, enhance treatment adherence, and increase life satisfaction.^[20]^ Accordingly, this study introduces the KAP model as a theoretical foundation to systematically investigate the relationship between fertility-related stress and marital adjustment in ART patients, providing both theoretical rationale and practical guidance for clinical interventions.

In summary, fertility stress has a considerable impact on the marital relationships of ART patients, yet current research remains limited in theoretical depth and interventional specificity. This retrospective study, grounded in the KAP model, enrolled 200 ART patients and collected data on demographics, fertility stress scores, marital adjustment, and KAP dimensions. It aimed to compare marital adjustment across different levels of fertility stress, analyze correlations, and identify influencing factors and population heterogeneity through multivariate linear regression and subgroup analysis. By elucidating the psychosocial mechanisms linking fertility stress and marital adaptation, this study seeks to inform more targeted marital support strategies during ART, ultimately promoting patients’ psychological well-being, marital satisfaction, and long-term treatment outcomes.

## 2. Methods

### 2.1. Study population and grouping

This study was approved by the Ethics Committee of West China Second University Hospital. This retrospective cross-sectional study included 200 patients who underwent ART treatment at our hospital between January 2023 and December 2024. Participants were stratified into a high-stress group (n = 100) and a low-stress group (n = 100) based on the median score of a fertility-related stress scale. Inclusion criteria were: age between 18 and 45 years, undergoing ART treatment, stable spousal relationship, and the ability to complete questionnaires independently. Exclusion criteria included a history of major psychiatric disorders, severe marital conflict, or absence of a spouse.

### 2.2. Data collection and scale assessment

Sociodemographic and clinical data – including age, height, weight, BMI, duration of marriage, educational level, and number of ART attempts – were collected via structured questionnaires. The Chinese version of the KAP scale was used to assess patients’ health behavior levels across 3 dimensions: knowledge, attitude, and practice. Fertility-related stress was evaluated using the Fertility Stress Scale, which has been previously applied in Chinese ART populations, showing good internal consistency (Cronbach α = 0.87) and construct validity. Marital adjustment was measured using the Chinese version of the Marital Adjustment Scale, which was translated and back-translated according to standard cross-cultural adaptation procedures, and has been validated in Chinese clinical populations, with Cronbach αcoefficients ranging from 0.82 to 0.90 across subscales. In the present study, both scales also demonstrated satisfactory internal consistency (Cronbach αvalues >0.80). The marital adjustment scale covers subdomains such as marital satisfaction, communication quality, and emotional support.

### 2.3. Statistical analysis

Statistical analysis was performed using SPSS version 26.0 (Chicago). Continuous variables were expressed as mean ± standard deviation and compared using independent sample t-tests. Categorical variables were presented as frequencies and percentages and compared using the chi-square test. Pearson correlation analysis was conducted to examine the association between fertility-related stress and marital adjustment. A multivariate linear regression model was constructed to identify independent predictors of marital adjustment, adjusting for potential confounders including age, BMI, educational level, and number of ART attempts. Prior to model fitting, multicollinearity among independent variables was examined using variance inflation factors, and no significant multicollinearity was detected (all variance inflation factors values < 2.0).

### 2.4. Subgroup analysis

To investigate the moderating effect of educational level on the relationship between fertility stress and marital adjustment, participants were stratified based on whether they had attained a bachelor’s degree or higher. Pearson correlation analyses were conducted separately within each subgroup to compare correlation strength across different educational backgrounds.

### 2.5. Data visualization and presentation

Key results were visualized using a series of charts and figures, including bar graphs for KAP dimension scores, comparison plots for marital adjustment subscales, scatter plots with fitted lines for the correlation between fertility stress and marital adjustment, and forest plots of regression coefficients. A 2-sided *P*-value of <.05 was considered the threshold for statistical significance. For results where the *P*-value was markedly smaller, we reported it as *P* < .001 to indicate a stronger level of evidence.

## 3. Result

### 3.1. Baseline characteristics comparison

A total of 200 patients undergoing ART were enrolled and divided into a high-stress group (n = 100) and a low-stress group (n = 100) according to the median fertility stress score. As shown in Table 1, there were no statistically significant differences between the 2 groups across demographic and clinical characteristics, including age, duration of marriage, education level, anthropometric indices, vital signs, lifestyle factors, and initial ART attempts, indicating good baseline comparability.

**Table 1:** Baseline characteristics of patients undergoing assisted reproductive technology by stress level.

Characteristic	High-stress group (n = 100)	Low-stress group (n = 100)	*t*/χ²	*P*-value
Age (year)	31.4 ± 4.2	30.9 ± 4.5	1.02	.309
Height (cm)	162.3 ± 5.8	161.7 ± 6.1	0.78	.436
Weight (kg)	57.2 ± 7.9	56.5 ± 8.3	0.68	.497
BMI (kg/m²)	22.1 ± 2.8	21.8 ± 3.0	0.78	.436
Resting heart rate (beats/min)	72.5 ± 8.7	71.8 ± 9.1	0.57	.569
Systolic blood pressure (mm Hg)	118.4 ± 12.6	117.1 ± 13.2	0.78	.436
Diastolic blood pressure (mm Hg)	75.8 ± 8.9	74.9 ± 9.3	0.64	.523
Smoking history positive rate (%)	12.00%	10.00%	0.26	.61
Fasting blood glucose (mmol/L)	5.2 ± 0.7	5.1 ± 0.6	1.22	.224
Number of initial ART attempts (times)	1.8 ± 0.9	1.7 ± 0.8	0.85	.396
Marriage duration (year)	5.2 ± 2.1	5.0 ± 2.3	0.6	.549
Education level “Bachelor’s degree or above” (%)	60.00%	63.00%	0.18	.671

### 3.2. Comparison of KAP model dimension scores

**Figure 1.**
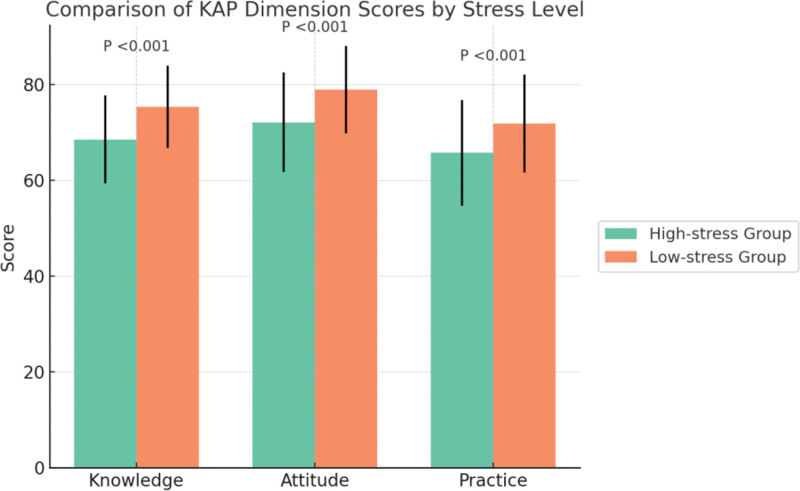
KAP model dimension scores by stress level. KAP = knowledge–attitude–practice.

In the KAP (Knowledge–Attitude–Practice) model assessment, the high-stress group scored significantly lower than the low-stress group across all 3 dimensions. Specifically, knowledge scores were 68.5 ± 9.2 vs 75.3 ± 8.6 (t = 6.06, *P* < .001), attitude scores were 72.1 ± 10.4 vs 78.9 ± 9.1 (t = 4.91, *P* < .001), and practice scores were 65.7 ± 11.0 vs 71.8 ± 10.2 (t = 4.00, *P* < .001). These results, as shown in Table 2 and Figure 1, indicate that patients experiencing higher fertility-related stress exhibited significantly lower levels of self-management in terms of knowledge acquisition, positive attitude formation, and behavioral practice.

**Table 2:** Comparison of KAP model dimension scores between high-stress and low-stress groups.

Dimension	High-stress group (mean ± SD)	Low-stress group (mean ± SD)	*t*	*P*-value
Knowledge	68.5 ± 9.2	75.3 ± 8.6	6.06	<.001
Attitude	72.1 ± 10.4	78.9 ± 9.1	4.91	<.001
Practice	65.7 ± 11.0	71.8 ± 10.2	4	<.001

KAP = knowledge–attitude–practice, SD = standard deviation.

### 3.3. Comparison of marital adjustment

**Figure 2.**
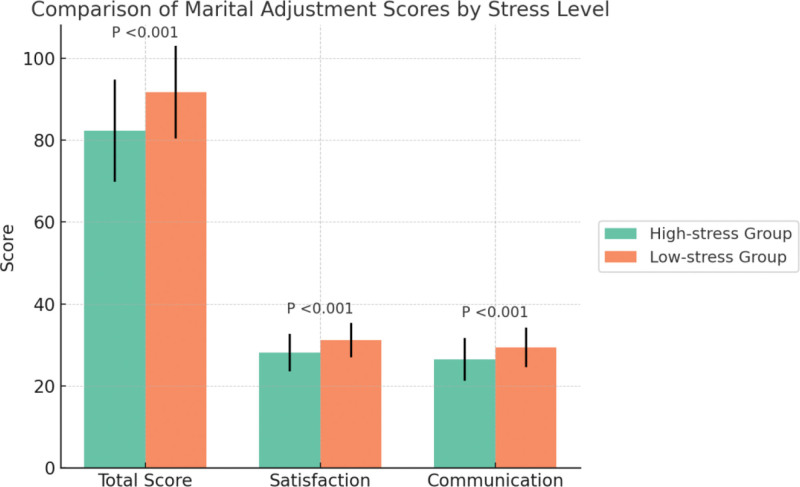
Comparison of marital adjustment scores by stress level.

Significant differences were observed between the high-stress and low-stress groups in terms of total marital adjustment scores and key subscale scores. The total marital adjustment score was significantly lower in the high-stress group compared to the low-stress group (82.3 ± 12.5 vs 91.7 ± 11.3; t = 6.25, *P* < .001). Specifically, the marital satisfaction subscale score was 28.1 ± 4.6 in the high-stress group versus 31.2 ± 4.2 in the low-stress group (t = 5.17, *P* < .001), while the communication quality subscale score was 26.5 ± 5.2 versus 29.4 ± 4.8, respectively (t = 4.76, *P* < .001), as shown in Figure 2. These findings suggest that patients experiencing higher fertility-related stress tend to have significantly lower levels of marital satisfaction and communication quality.

### 3.4. Correlation analysis between fertility-related stress and marital adjustment

**Figure 3.**
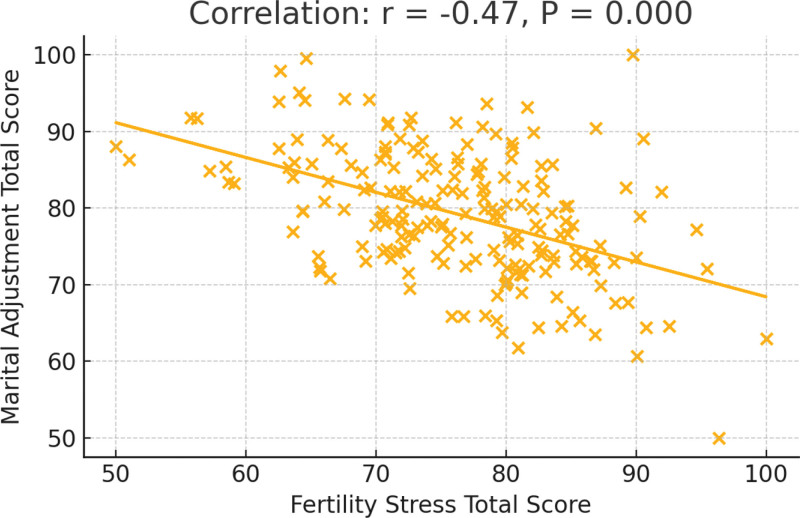
Scatter plot of fertility-related stress total score versus marital adjustment total score.

Pearson correlation analysis revealed a moderate and statistically significant negative correlation between the total fertility-related stress score and the total marital adjustment score (*r* = –0.48, *P* < .001). As shown in Figure 3, the scatter plot with a fitted regression line illustrates that, among the 200 participants, higher fertility-related stress scores were associated with lower marital adjustment scores.

### 3.5. Multiple linear regression analysis

**Figure 4.**
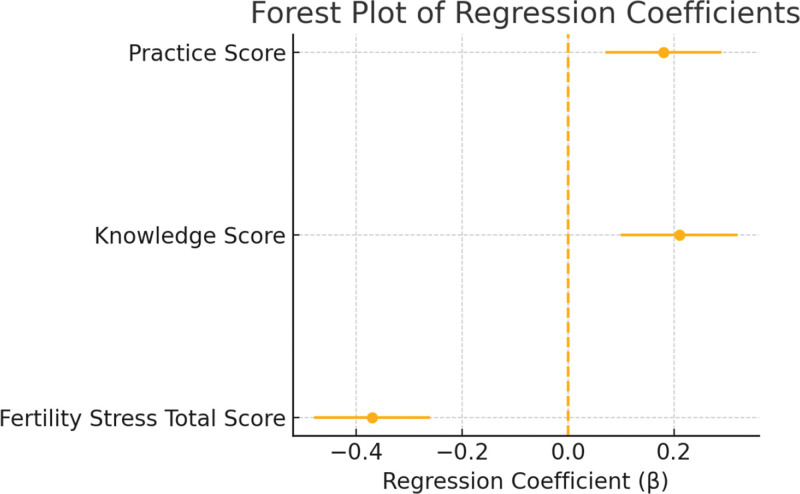
Forest plot of regression coefficients.

After adjusting for confounding variables including age, BMI, education level, and number of initial ART attempts, multiple linear regression analysis demonstrated that the total fertility-related stress score remained an independent negative predictor of the total marital adjustment score (β = –0.37, 95% CI:–0.48 to–0.26, *P* < .001). Meanwhile, the knowledge dimension score (β = 0.21, 95% CI: 0.10–0.32, *P* < .001) and the practice dimension score (β = 0.18, 95% CI: 0.07–0.29, *P* = .002) from the KAP model were identified as significant positive predictors of marital adjustment. The regression coefficients, 95% confidence intervals, and P-values for each variable are presented in the table below. Figure 4 shows the corresponding forest plot, where the vertical dashed line represents the null effect (β = 0), and the dots and horizontal lines indicate the estimated coefficients and their confidence intervals, respectively.

### 3.6. Subgroup analysis: influence of educational level

Pearson correlation analysis stratified by educational level revealed that the negative correlation between total fertility-related stress and total marital adjustment was stronger among participants with a bachelor’s degree or higher (*r* = –0.52, *P* < .001) compared to those with an associate degree or below (*r* = –0.39, *P* < .001). This finding suggests that individuals with higher education levels may be more susceptible to the adverse impact of fertility-related stress on marital adjustment.

Table 3 summarizes the correlation coefficients and corresponding *P*-values within each educational subgroup.

**Table 3:** Subgroup analysis of correlation between fertility-related stress and marital adjustment by education level.

Education level	Pearson r	*P*-value
Bachelor’s or higher	–0.52	<.001
Associate degree or below	–0.39	<.001

## 4. Discussion

With the widespread application of ART, an increasing number of infertile couples are realizing their reproductive goals through medical interventions such as in vitro fertilization and artificial insemination.^[21]^ However, the high cost, uncertain outcomes, and repeated treatment failures in the ART process often impose substantial psychological burdens on patients, leading to elevated fertility-related stress. This stress can adversely affect marital relationships and family stability.^[22]^ Marital adjustment, as a key indicator of relationship quality between partners, is therefore worthy of further investigation in ART patients. Based on the KAP theoretical model, this study systematically evaluated the association between fertility-related stress, cognitive-behavioral characteristics, and marital adjustment in ART patients, aiming to provide a theoretical foundation for psychological interventions and marital support strategies.

First, baseline characteristics between the high-stress and low-stress groups were comparable in terms of age, marital duration, education level, height, weight, BMI, vital signs, smoking history, fasting glucose, and number of ART attempts, suggesting the groups were well matched and free from major confounding by demographic or clinical factors.

In terms of KAP dimension scores, patients in the high-stress group scored significantly lower in knowledge, attitude, and practice compared to those in the low-stress group. This indicates that fertility-related stress is not only a psychological burden but may also hinder patients’ acquisition of ART-related knowledge, the formation of positive attitudes, and the implementation of healthy behaviors. These findings are consistent with previous studies conducted among Chinese infertile women, which identified limited knowledge and cognitive biases as important contributors to elevated fertility stress.^[23]^ Our study further quantified each KAP dimension and compared them across stress levels, thereby offering specific targets for future cognitive-behavioral interventions and expanding upon the existing literature.

Regarding marital adjustment, the high-stress group had significantly lower total scores, particularly in the subscales of marital satisfaction and communication quality. This aligns with prior research indicating that ART-related emotional fluctuations and communication difficulties are associated with decreased marital satisfaction among patients undergoing fertility treatment.^[24]^ By quantitatively grouping participants by stress level, our study confirmed the disadvantages in marital adjustment among high-stress individuals and, through the lens of the KAP model, explored potential psychological-behavioral mechanisms, laying a foundation for future intervention studies.

Pearson correlation analysis showed a moderate negative correlation between total fertility-related stress and total marital adjustment, suggesting that as stress levels increase, marital adjustment decreases. This inverse relationship also manifested in key subdomains such as marital satisfaction and emotional support, indicating that fertility stress may compromise emotional intimacy and satisfaction, thereby weakening marital adaptability.

Further, in multivariate linear regression analysis controlling for age, BMI, education level, and number of ART attempts, fertility-related stress remained an independent negative predictor of marital adjustment (β = –0.37, *P* < .001). Meanwhile, knowledge (β = 0.21, *P* < .001) and practice (β = 0.18, *P* = .002) dimensions of the KAP model emerged as significant positive predictors. These findings suggest that enhancing patients’ knowledge and behavioral capacity regarding fertility treatment may reduce stress and improve marital relationships. This is consistent with other studies showing that cognitive-behavioral interventions can alleviate intra-family conflict and psychological distress in ART patients.^[25]^

Subgroup analysis revealed a stronger negative correlation between fertility-related stress and marital adjustment among patients with a bachelor’s degree or higher (*r* = –0.52) compared to those with an associate degree or below (*r* = –0.39). This finding suggests that individuals with higher education levels may not only have elevated expectations regarding treatment outcomes but also exhibit psychological characteristics that exacerbate vulnerability to stress. Previous studies have shown that highly educated patients place a stronger emphasis on maintaining a sense of control, are more prone to information overload when facing complex medical decisions, and tend to adopt negative coping strategies such as avoidance or self-blame in the context of fertility stress.^[24,25]^ These factors may partly explain their greater susceptibility to stress spillover into the marital domain. This important observation, seldom addressed in prior studies, represents another novel contribution of our research.

The strengths of this study include: a moderate sample size and use of a median split to mitigate the impact of outliers; the application of the KAP model to evaluate psychological-behavioral traits, providing concrete targets for clinical intervention; the use of both regression and subgroup analyses to examine the pathways linking stress and marital adjustment and to capture population heterogeneity; and filling a gap in domestic literature regarding the systematic evaluation of marital adjustment and fertility stress in ART patients.

However, several limitations should be acknowledged. First, the retrospective cross-sectional design precludes causal inference; although associations were identified, the temporal direction of effects cannot be determined. Future longitudinal and interventional studies are needed to verify the causal pathways. Second, as a single-center study, the findings may be influenced by regional or cultural factors, limiting their generalizability. Third, the assessment of marital adjustment relied on self-reported questionnaires, which may be subject to reporting bias; future research should incorporate partner evaluations or adopt multi-informant and longitudinal designs to strengthen validity.

In conclusion, this study confirms a significant association between fertility-related stress and marital adjustment in ART patients. Higher stress levels were associated with poorer marital adaptation, whereas knowledge and practice dimensions were positively related to better outcomes. This negative impact was particularly pronounced among highly educated individuals. From a clinical perspective, KAP-based cognitive-behavioral interventions – such as structured health education, behavioral skills training, and peer-support programs – may help patients acquire accurate knowledge, adopt positive attitudes, and improve coping behaviors. These targeted measures could alleviate stress, enhance marital adjustment, and ultimately contribute to better treatment adherence and reproductive outcomes.

## 5. Conclusion

This study, based on the KAP model, confirmed that fertility-related stress is closely associated with poorer marital adjustment among patients undergoing ART, while knowledge and practice dimensions act as protective factors.

From a clinical perspective, these findings highlight the need to go beyond symptom observation and actively integrate psychological interventions into ART care. Tailored KAP-based support – such as structured health education, behavioral skills training, and peer-support programs – may effectively reduce stress and strengthen marital adjustment. In particular, highly educated patients, who appear more vulnerable to the adverse effects of fertility stress, could benefit from targeted strategies that address their higher expectations, information demands, and coping styles.

Although limited by its cross-sectional design, this study provides a theoretical foundation for future longitudinal and interventional research to validate causal pathways and develop evidence-based psychosocial support protocols in ART settings.

## Author contributions

**Conceptualization:** Limin He, Qian Yang, Lihua Zhou.

**Data curation:** Limin He, Lihua Zhou.

**Formal analysis:** Qian Yang, Lihua Zhou.

**Investigation:** Lian Liu, Lihua Zhou.

**Methodology:** Qian Yang, Lihua Zhou.

**Validation:** Lian Liu, Lihua Zhou.

**Visualization:** Lihua Zhou.

**Writing – original draft:** Limin He, Lihua Zhou.

**Writing – review & editing:** Limin He, Lihua Zhou.
